# P-2072. COVID-19 and RSV Prevention Products in Pregnancy – Overcoming COVID-19 Network, United States, October 2023–March 2024

**DOI:** 10.1093/ofid/ofae631.2228

**Published:** 2025-01-29

**Authors:** Regina Simeone, Margaret M Newhams, Laura D Zambrano, Amber Orzel, Megan Lindley, Amanda B Payne, Jemima Calixte, Katherine N Lindsey, Michael J Wu, Adrienne G Randolph, Angela P Campbell

**Affiliations:** CDC, Atlanta, Georgia; Boston Children's Hospital, Boston, Massachusetts; Centers for Disease Control and Prevention, Atlanta, GA; Boston Children's Hospital, Boston, Massachusetts; Centers for Disease Control & Prevention, Atlanta, GA; CDC, Atlanta, Georgia; Boston Childrens Hospital, Boston, Massachusetts; Centers for Disease Control and Prevention, Atlanta, GA; Centers for Disease Control and Prevention, Atlanta, GA; Boston Children's Hospital, Harvard Medical School, Boston, Massachusetts; Centers for Disease Control and Prevention, Atlanta, GA

## Abstract

**Background:**

Maternal vaccines for COVID-19 and respiratory syncytial virus (RSV) are tools to prevent severe infant respiratory disease. Among recently pregnant people with hospitalized infants < 6 months with COVID-19 or COVID-like illness, we evaluated receipt of these products and association with provider recommendations, other vaccine receipt, and acceptability of future vaccines in pregnancy.

**Figure:**
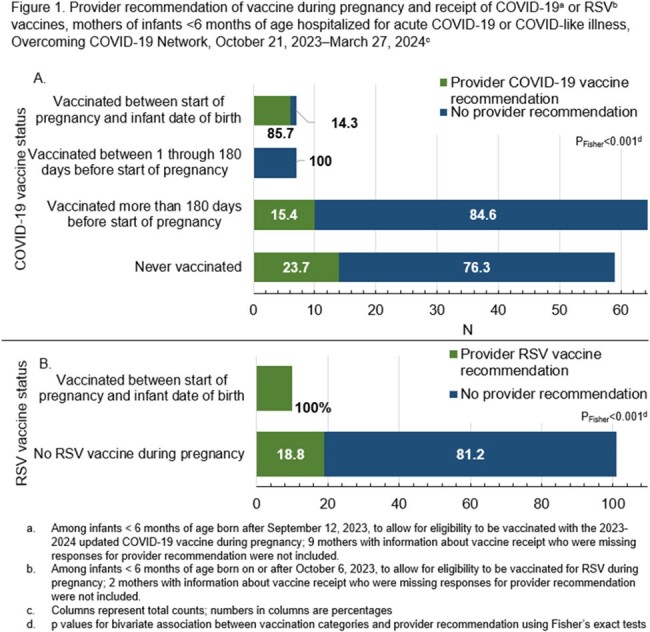

**Methods:**

Mothers of infants < 6 months hospitalized either for COVID-19 or COVID-like illness were interviewed for the Overcoming COVID-19 Network. Outcomes were 1) verified receipt of COVID-19 and maternal report of RSV vaccines during pregnancy by provider recommendation (among those eligible for updated/new vaccine based on date of vaccine approval and infant date of birth), 2) influenza and Tdap vaccine receipt during pregnancy, and 3) willingness to receive vaccines in future pregnancies.

**Figure:**
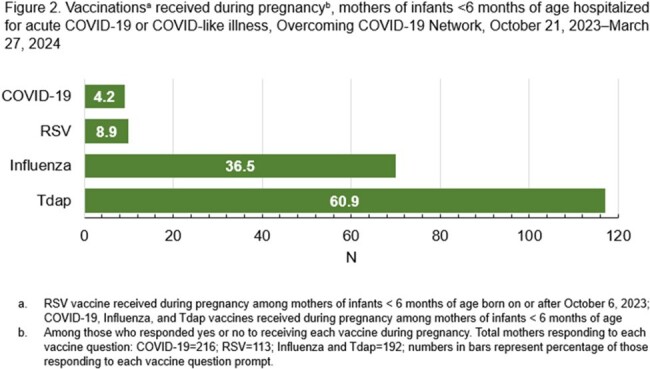

**Results:**

Caregivers of 514 (68%) of 760 hospitalized infants < 6 months were interviewed; 216 (42%) of 514 interviews were among mothers. Seven (5%) of 147 eligible mothers received COVID-19 vaccine during pregnancy, 8 (5%) were vaccinated 1 to 180 days before pregnancy, and 132 (90%) were vaccinated >180 days before pregnancy or not at all. Ten (9%) of 113 eligible mothers received the RSV vaccine during pregnancy; 20 (19%) mothers who did not receive RSV vaccine reported their infant receiving an infant monoclonal antibody against RSV. Provider recommendation was associated with maternal receipt of both COVID-19 and RSV vaccines during pregnancy (Figure 1A, 1B). Tdap was the most received vaccine during pregnancy (n=117 [61%]) (Figure 2). Willingness to receive vaccines in a future pregnancy was common for Tdap (n=93, 58%), influenza (n=88, 55%), and RSV (n=87, 54%). Only 22% (n=35) of mothers stated they would be willing to receive a COVID-19 vaccine in a future pregnancy.

**Conclusion:**

Among mothers of infants hospitalized with COVID-19 or COVID-19-like illness, few had received a COVID-19 vaccine during or within 180 days before pregnancy. Tdap and influenza vaccines were more commonly received during pregnancy and were more acceptable in future pregnancies. Provider recommendation was associated with receipt of COVID-19 and RSV vaccines during pregnancy.

**Disclosures:**

Regina Simeone, PhD, Pfizer: Stocks/Bonds (Private Company) Adrienne G. Randolph, MD, MSc, Illumina, Inc.: Reagents|Inotrem: Advisor/Consultant|Thermo Fisher, Inc.: Advisor/Consultant

